# Axotomy-Induced miR-21 Promotes Axon Growth in Adult Dorsal Root Ganglion Neurons

**DOI:** 10.1371/journal.pone.0023423

**Published:** 2011-08-10

**Authors:** Iain T. Strickland, Louise Richards, Fiona E. Holmes, David Wynick, James B. Uney, Liang-Fong Wong

**Affiliations:** 1 School of Clinical Sciences, University of Bristol, Bristol, United Kingdom; 2 School of Physiology and Pharmacology, University of Bristol, Bristol, United Kingdom; Nathan Kline Institute and New York University, United States of America

## Abstract

Following injury, dorsal root ganglion (DRG) neurons undergo transcriptional changes so as to adopt phenotypic changes that promote cell survival and axonal regeneration. Here we used a microarray approach to profile changes in a population of small noncoding RNAs known as microRNAs (miRNAs) in the L4 and L5 DRG following sciatic nerve transection. Results showed that 20 miRNA transcripts displayed a significant change in expression levels, with 8 miRNAs transcripts being altered by more than 1.5-fold. Using quantitative reverse transcription PCR, we demonstrated that one of these miRNAs, miR-21, was upregulated by 7-fold in the DRG at 7 days post-axotomy. In dissociated adult rat DRG neurons lentiviral vector-mediated overexpression of miR-21 promoted neurite outgrowth on a reduced laminin substrate. miR-21 directly downregulated expression of Sprouty2 protein, as confirmed by Western blot analysis and 3′ untranslated region (UTR) luciferase assays. Our data show that miR-21 is an axotomy-induced miRNA that enhances axon growth, and suggest that miRNAs are important players in regulating growth pathways following peripheral nerve injury.

## Introduction

Sciatic nerve injury triggers gene expression changes in the dorsal root ganglion (DRG) of transected nerves and in the microenvironment of the nerve stumps. These transcriptional alterations translate into phenotypic changes that enable the damaged DRG neurons to adapt to the injury, for example by promoting stress response and cell survival pathways as well as growth programs to regenerate severed axons.

In order to elucidate the molecular pathways that contribute to neural regeneration a number of laboratories have undertaken microarray and proteomic approaches to identify differentially expressed genes and proteins in DRG neurons following nerve axotomy [1]–[9]. A large number of genes and proteins were found to be regulated; these were diverse and distinct, comprising members of several classes such as neuropeptides, receptors, ion channels, signal transduction molecules, synaptic vesicle proteins, cell cytoskeletal components, extracellular matrices and inflammatory mediators. While these studies have provided an insight into the molecular changes that occur in the injured nerve and its environs, it is still unknown how these global changes are regulated in a coordinated fashion. One possibility is transcriptional regulation by signal transduction molecules or transcription factors such as cAMP [10], [11], c-Jun [12] or retinoic acid receptor β2 [13], [14]. Another possible mechanism of coordinated control can occur at the post-transcriptional level, for example regulation by microRNAs (miRNAs).

miRNAs have recently emerged as important post-transcriptional regulators in several developmental and physiological processes. In the nervous system, miRNAs have been implicated in cell specification [15], [16], neurite outgrowth [17], dendritic spine development [18]–[20] and disease [21], [22]. More recently it has been demonstrated that abolition of the miRNA pathway in the Nav1.8 population of nociceptive neurons attenuated inflammatory pain [23]. We postulated that altered miRNA levels after peripheral nerve injury can contribute to growth programs that promote axonal regeneration. Here we show that an axotomy-regulated miRNA, miR-21, promotes neurite growth from injured adult DRG neurons by targeting the Sprouty2 protein. Our results uncover a role for miRNAs in regulating axonal regeneration following peripheral nerve injury.

## Results

### miRNA regulation after sciatic nerve transection

**Figure 1:**
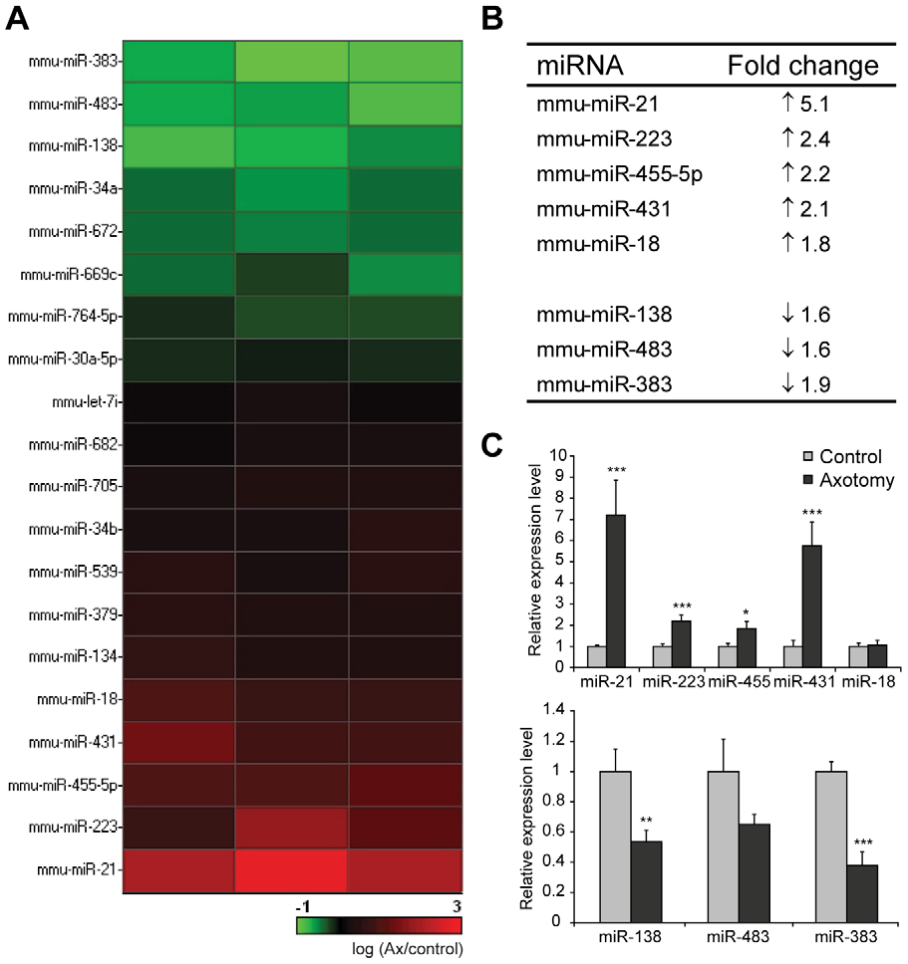
Expression profiling of miRNAs in the DRG following sciatic nerve injury. (a) miRNAs that were significantly altered at 7 days after sciatic nerve injury in three different experiments. Colour coding: control versus axotomised DRG on a logarithmic scale; green represents decreased level while red represents increased level in axotomised DRG (n = 3). (b) miRNAs that displayed at least 1.5 fold change in expression after axotomy. (c) The miRNA changes were validated by quantitative real-time PCR. miRNA expression was normalised to that of the U6B small nuclear RNA gene (RNU6B). Asterisks indicate significant differences in axotomised DRGs compared to controls. *** *p*<0.001, ** *p*<0.01, * *p*<0.05, Students' t-test, n = 4.

We carried out a microarray screen to determine miRNA changes in adult rodent DRG after sciatic nerve injury. After transection, injured peripheral nerves initially undergo Wallerian degeneration before regrowing. The timepoint of 7 days post-axotomy was chosen to capture miRNA expression profiles at a time when the injured neurons were beginning to regenerate. Total RNA was extracted from axotomised and control contralateral DRGs and simultaneously hybridised to microarrays that contained probes from all mouse mature miRNAs listed in the Sanger database (Sanger version 9.0). Statistical analyses revealed that 20 miRNA transcripts were differentially expressed in axotomised DRG compared to the non-axotomised contralateral DRG; with 8 being upregulated and 12 down-regulated (*p*<0.05; Fig. 1A, Table S1). Using a 1.5-fold cut-off, 8 candidate miRNAs remained for further investigation (Fig. 1B). miR-21, miR-223, miR-455-5p, miR-431 and miR-18 were significantly increased, while miR-138, miR-483 and miR-383 were significantly decreased following nerve transection. These observed changes were further validated by quantitative real-time reverse transcription PCR (qRT-PCR) and from these candidates we chose to further examine miR-21 (Fig. 1C).

**Figure 2:**
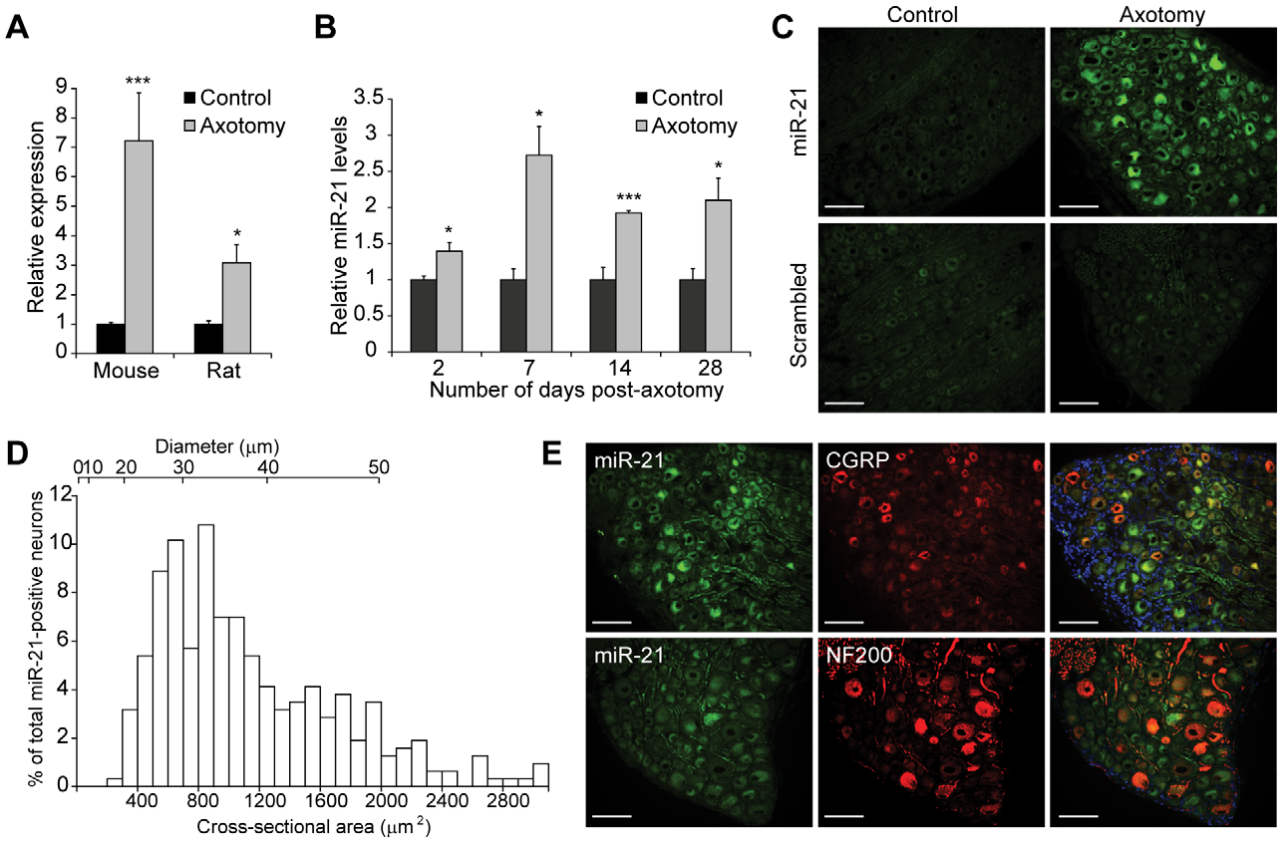
miR-21 upregulation in the DRG following sciatic nerve injury. (a) Relative miR-21 expression in mouse and rat DRG at 7 days following axotomy. Expression of miR-21 was normalised to that of the U6B small nuclear RNA gene (RNU6B). Asterisks indicate significant differences in axotomised DRGs compared to controls. *** *p*<0.001, * *p*<0.05, Students' t-test, n = 4. (b) Relative miR-21 expression in axotomised rat DRG at various timepoints after injury, normalised to that of the U6B small nuclear RNA gene (RNU6B). Asterisks indicate significant differences in axotomised DRGs compared to controls. *** *p*<0.001, * *p*<0.05, Students' t-test, n = 3 at each timepoint. (c) *In situ* hybridisation studies with miR-21 and control scrambled probes show upregulation of miR-21 following axotomy and miR-21 localisation in DRG neurons (n = 3). Scale bar represents 100 µm. (d) Size profiling of miR-21 expressing neurons in the DRG. (e) Co-localisation of miR-21 (green) with neurofilament 200 (NF200) and calcitonin-G related peptide (CGRP) markers (in red). Blue represents DAPI staining. Scale bar represents 100 µm.

Following axotomy, miR-21 expression increased 7-fold and 3-fold in the mouse and rat DRG respectively (Fig. 2A), indicating that injury-induced upregulation of miR-21 was replicated in both rodent models. qRT-PCR analysis also indicated that miR-21 increased significantly as early as 2 days post-injury, which was sustained 28 days post-injury (Fig. 2B). *In situ* hybridisation studies confirmed that the upregulation of miR-21 occurred in rat DRG neurons at 7 days post-injury (Fig. 2C). Analysis of miR-21 neuronal profiles indicated that increased miR-21 expression occurred in neurons of all sizes, with 29.0 ± 4.1%, 40.5 ± 4.2% and 29.6 ± 2.6% of all miR-21 expressing neurons found to be in small (<30 µm), medium (30–40 µm) and large (>40 µm) diameter neurons respectively (Fig. 2D). Furthermore, co-localisation experiments demonstrated that miR-21 was detected in large diameter neurons expressing neurofilament 200kD (NF200) as well as small and medium diameter neurons expressing Calcitonin gene-related peptide (CGRP, Fig. 2E).

### miR-21 increases neurite outgrowth from DRG neurons

**Figure 3:**
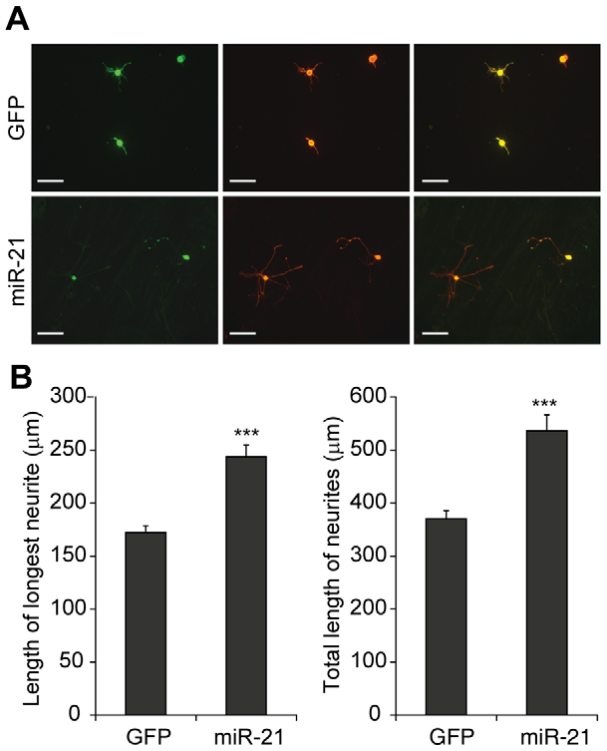
miR-21 increased neurite outgrowth in DRG neurons. (a) Neurite outgrowth (detected by βIII tubulin in red) observed in GFP or miR-21 transduced DRG neurons (green). Scale bar represents 100 µm. (b) miR-21 increased the mean length of the longest neurite as well as the mean total neurite length (per neuron) compared to GFP control. Asterisks indicate significant differences in miR-21 neurons compared to GFP controls. *** *p*<0.001, Students' t-test, n = 3.

In order to determine the effects of miR-21 on neuronal growth, we overexpressed miR-21 in dissociated adult rat DRG neurons that were plated on a reduced laminin substrate (0.1 µg/ml). Increased miR-21 levels in these neurons were achieved by transduction with GFP-tagged lentiviral vectors that were verified for mature miR-21 overexpression using real-time PCR analysis (Fig. S1). In DRG cultures transduced with control GFP lentiviral vectors, neurons extended either very short neurites or no neurites at all (Fig. 3A). In contrast miR-21 overexpressing neurons showed a significant increase in neurite outgrowth (Fig. 3A). Quantification of fibre outgrowths from transduced neurons indicated that miR-21 significantly increased the mean length of the longest neurite compared to GFP (244 ± 10.9 µm vs 172 ± 6.4 µm, *p*<0.001; Fig. 3B). In addition, miR-21 increased the mean total neurite length (per neuron) in the DRG cultures compared to GFP control (537 ± 30.1 µm vs 370 ± 15.3 µm, *p*<0.001; Fig. 3B). These data demonstrate that overexpression of miR-21 promoted outgrowth in adult rat DRG neurons by increasing neurite extension.

### miR-21 targets SPRY2 expression

**Figure 4:**
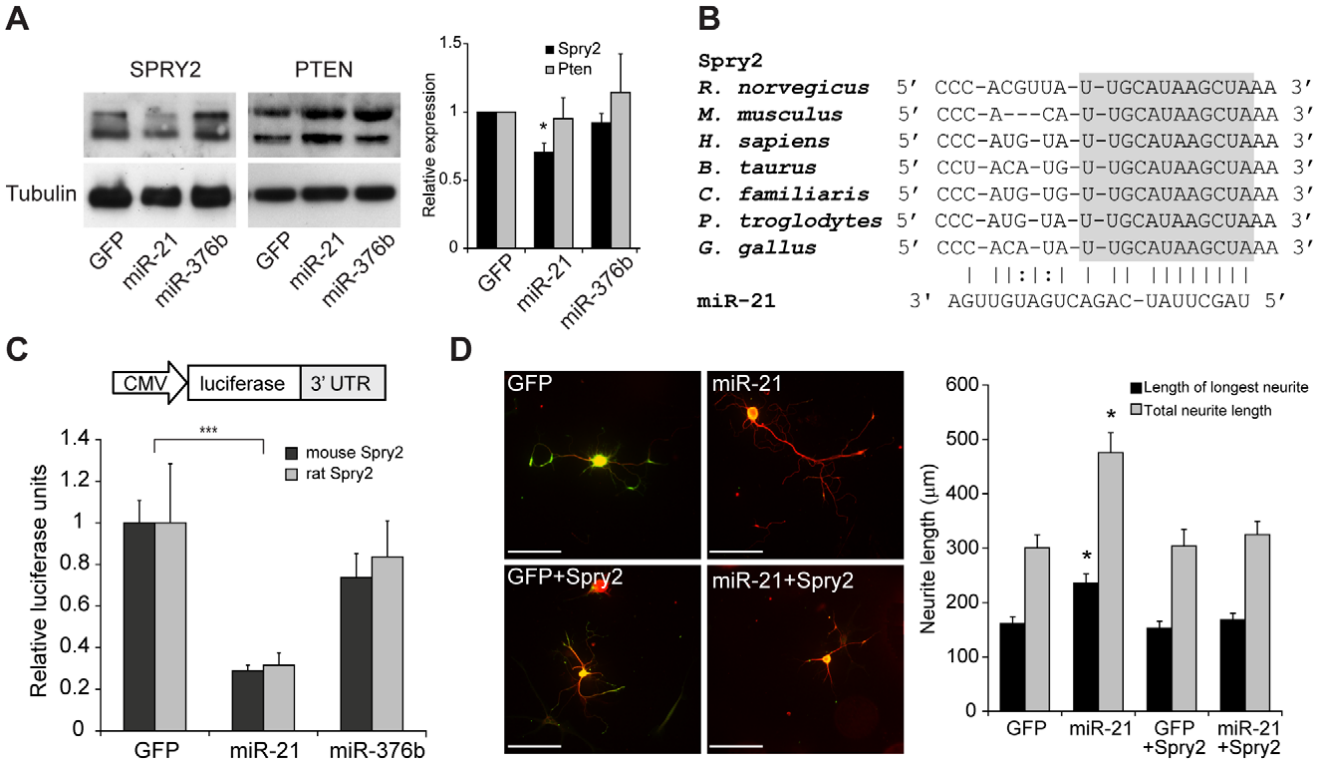
miR-21 targets SPRY2 protein. (a) Western blot analysis to detect SPRY2 and PTEN protein in DRG neurons transduced with GFP, miR-21 or miR-376b (control) lentiviral vectors. Fold changes in protein expression are normalised to α-tubulin and expressed as a fraction of the levels in GFP treated cells, which are assigned a value of 1.00. Fold changes in SPRY2 and PTEN are reflected in bar chart. Asterisk indicates significant difference in miR-21 neurons compared to miR-376b controls. * *p*<0.05, Students' t-test, n≥3. (b) Alignment of the 3′ UTR sequence of SPRY2 in 7 species with miR-21, highlighting the fully conserved sequence in grey. (c) Relative luciferase expression in mouse (black) and rat (grey) SPRY2 luciferase reporters cotransfected with miRNA-expressing constructs in HeLa cells. Asterisks indicate significant difference in miR-21 neurons compared to controls. ***p<0.001, Students' t-test, n = 4. (d) Neurite outgrowth (detected by βIII tubulin in red) observed in GFP, miR-21, GFP+SPRY2 and miR-21+SPRY2 transduced DRG neurons (green). Scale bar represents 100 µm. miR-21 increased the mean length of the longest neurite as well as the mean total neurite length (per neuron). In the presence of SPRY2, miR-21-mediated increase in neurite outgrowth was abolished. Asterisks indicate significant difference in miR-21 neurons compared to GFP control. *** *p*<0.001, one-way ANOVA with Tukey's post test, n = 3.

Confirmed mRNA targets of miR-21 include PTEN, TPM1, PDCD4 and SPRY2 (reviewed in [24]). We sought to determine if miR-21 knocked down Sprouty 2 (SPRY2) or phosphatase and tensin homolog (PTEN) tumour suppressor protein levels in mature DRG neurons. Western blot analysis revealed a 30% decrease in the expression of endogenous SPRY2 compared to control transduced DRG neurons, whilst no significant change in PTEN protein levels was observed (*p*<0.05; Fig. 4A). Analysis by miRANDA target detection software (www.microrna.org) indicated that miR-21 has a high energy binding site in both the mouse and rat *Spry2* 3′ untranslated region (UTR), at 1520–1536 bp and 1430–1449 bp respectively. Furthermore, this miR-21 binding site appears to be highly conserved across several species, including the human *Spry2* (Fig. 4B). To show that miR-21 directly targeted SPRY2 we cloned the 3′ UTR of mouse and rat *Spry2* downstream of a luciferase reporter. Cotransfection of these luciferase reporter plasmids along with miR-21 resulted in significant decreases in luciferase activity of 71 ± 2.7% and 68 ± 6.0% compared to controls in the mouse and rat SPRY2 reporters respectively (*p*<0.05; Fig. 4C). Taken together the Western blot analysis and luciferase reporter assay data indicate that miR-21 binds directly in the 3′ UTR of both mouse and rat SPRY2 mRNA transcripts, and consequently downregulates SPRY2 protein expression in DRG neurons.

To further validate the role of SPRY2 in miR-21 mediated neurite outgrowth, we transduced rat DRG cultures with miR-21 and a lentiviral vector that overexpressed miR-21-insensitive Spry2 (rat Spry2 cDNA without the 3′ UTR) (Fig. S1). As described previously miR-21 overexpressing neurons demonstrated a significant increase in the mean length of the longest neurite and mean total neurite length (per neuron) compared to GFP controls (236 ± 16.5 µm vs 161 ± 12.8 µm and 476 ± 36.7 µm vs 301 ± 24.0 µm respectively; *p*<0.001, Fig. 4D). Transduction of miR-21 together with SPRY2 abrogated the growth promoting effects of miR-21 in DRG neurons, with the mean length of the longest neurite and mean total neurite length reduced to 168 ± 12.3 µm and 325 ± 24.2 µm respectively; Fig. 4D). These data demonstrated that SPRY2 is a key player in the neurite outgrowth response mediated by miR-21 in DRG neurons.

## Discussion

Sciatic nerve injury activates transcription factors such as c-Jun [25], ATF3 [26] and Stat3 [27], which in turn modulate gene expression and stimulate axon growth to reconnect with peripheral targets. Here we investigated if sciatic nerve injury induced changes in small noncoding RNAs that can regulate gene expression at the post-transcriptional level. Using a microarray screen we identified 8 miRNAs that were regulated after axotomy. We further show that miR-21 enhanced neurite outgrowth in adult rat DRG neurons. This, to our knowledge, is the first time a regenerative role for miR-21 in neurons has been demonstrated.

Of the 8 miRNAs that were regulated after axotomy, only miR-431 and miR-138 have been shown to be expressed in the central nervous system. miR-223 appears to be predominantly present in hematopoietic cells but interestingly has been shown to be highly expressed in neutrophils that are present in the spinal cord during the early phase of spinal cord injury [28]. miR-431 was found to be present in embryonic and postnatal mouse brains but levels were lower in adult mouse brains [29]. miR-383 is expressed in germ cells and has been shown to target interferon regulatory-factor 1 (IRF-1), a transcription factor that controls expression of genes related to inflammation and injury [30]. IRF-1 expression is increased in neurons following ischaemic injury [31], [32] and it is plausible that decreased miR-383 in the DRG following axotomy can modulate signalling cascades through IRF-1. miR-138 is interesting as it is highly enriched in the synapse and negatively regulates dendritic spine size by targeting the depalmitoylation enzyme acyl protein thioesterase 1 (APT1) [19]. Interestingly in the same paper, miR-21 also exhibited significant increased expression in rat synaptosomes compared to whole forebrain extract [19].

miR-21 is a commonly dysregulated miRNA in many forms of cancer and cardiovascular disease (reviewed in [24], [33]) however its function in the nervous system has not been examined. We showed that miR-21 was significantly increased in the DRG 2 days after axotomy, and that this increase was sustained up to 28 days post-injury. The persistent upregulation of miR-21 expression may serve to maintain the intrinsic growth capacity of the injured neuron for the long period of time required for axons to regenerate the considerable distance to reinnervate peripheral targets. Indeed miR-21 was observed to be upregulated in the DRG at 6 months after injury in a sciatic nerve denervation model [34]. Activation of miR-21 expression is possibly mediated through IL-6/STAT3 response elements present in the *miR-21* gene [35]. IL-6 is significantly upregulated in the DRG after sciatic nerve transection [36] and has been shown to play an important role in the conditioning lesion response of the DRG that can facilitate regeneration in the central nervous system [37], [38]. IL-6 signals through the Janus-kinase (JAK)-signal transducer and activator of transcription (STAT) signalling pathway and phosphorylated pSTAT3, one of the members of the STAT family of transcription factors, has also been shown to be significantly upregulated in the DRG as early as 6 hours after sciatic axotomy [36], [39]. The timing of IL6/pSTAT3 expression in the DRG correlates well with increased miR-21 levels and future experiments will determine that this is indeed the mechanism by which miR-21 expression is activated by nerve injury.

Studies have shown that miR-21 regulates PTEN tumour suppressor in hepatocellular cancer cells [40] and SPRY2 in cardiomyocytes [41]. These targets were of interest as inhibition of PTEN and SPRY2 increased neurite outgrowth of sensory axons *in vitro* [42], [43]. In DRG neurons, we observed miR-21-induced knockdown of SPRY2 but not PTEN. We further confirmed that miR-21 directly bound to the SPRY2 3′ UTR region using luciferase reporter assays. Based on these observations, we postulate that the functional significance of axotomy induced miR-21 levels is to subtly modulate SPRY2 protein levels in order to promote neurite outgrowth from the injured DRG neurons. In the presence of miR-21-insensitive SPRY2, the growth promoting effects of miR-21 in DRG neurons were abolished, indicating that SPRY2 is an important player in the axonal outgrowth response following miR-21 overexpression. Sprouty proteins were first discovered as antagonists of the fibroblast growth factor (FGF) signalling pathway in *Drosophila* tracheal development [44]. Of the 4 Sprouty isoforms SPRY2 expression is the highest in the DRG [43] and has been shown to inhibit neurite outgrowth in PC12 cells and cerebellar granule neurons [45], [46]. Furthermore, shRNA-mediated downregulation of SPRY2 promoted axon growth by DRG neurons [43]. One prediction from our data would be that SPRY2 levels in the DRG should change following sciatic nerve injury. To our knowledge, changes in SPRY2 expression have not been described in published microarray and proteomics datasets. However, Hausott *et al.* [43] have observed that there was no change in Spry2 mRNA levels in the axotomised DRG following sciatic nerve transection using qPCR detection methods. The lack of a detectable change in SPRY2 mRNA levels does not preclude a change in SPRY2 protein concentration in DRG neurons, as miRNAs act post-transcriptionally to modulate protein levels.

Sprouty proteins specifically inhibit the Ras/Raf/ERK pathway and downregulation of SPRY2 increased phospho-ERK1/2 [41]. The increased pERK can in turn induce functional changes by phosphorylating kinases, receptors, ion channels and transcription factors, thereby promoting growth programs in the injured DRG neuron. Unfortunately we were unable to detect a statistically significant change in pERK1/2 levels in miR-21 overexpressing DRG neurons and further work will be required to determine if pERK signalling is indeed involved in mediating the neurite outgrowth response caused by miR-21. It is also unlikely that SPRY2 is the sole target of miR-21 in DRG neurons and further identification of functional miR-21 targets in DRG neurons will allow us to elucidate other yet unidentified pathways by which miR-21 can promote axonal regeneration.

The sequences of both precursor and mature forms of miR-21 are extremely well conserved across species, suggesting a fundamental function for miR-21 in physiological processes. Our study demonstrates that miR-21 transcripts are physiologically regulated by peripheral nerve injury. Increased miR-21 levels enhanced neurite outgrowth from DRG neurons by targeting the SPRY2 protein, suggesting that axotomy-induced miR-21 plays a role in promoting axonal regeneration following peripheral nerve injury. It is clear that miRNAs are important players in a complex network of regulators that modulate growth processes and there is no doubt that roles for other miRNAs in peripheral nerve injury paradigms will be revealed in the future. Further elucidation of the contribution of miRNAs to axonal regeneration and their specific targets may uncover novel targets for drug development for the treatment of nerve injury.

## Materials and Methods

### Sciatic nerve injury

All mouse and rat experiments were approved by the University of Bristol Ethical Review Group and carried out in accordance with the UK Animals (Scientific Procedures) Act. Animals were maintained on a 12-h/12-h light/dark cycle in a room with controlled temperature and humidity with *ad libitum* access to food and water. Animals (C57/Bl mice or adult Wistar rats) were anesthetized with an intraperitoneal (i.p.) injection of ketamine (60 mg/kg) and medetomidine (0.25 mg/kg). The left sciatic nerve was exposed at mid-thigh level and gently dissected out from the surrounding connective tissue. The nerve was ligated at approximately 1cm proximal to the nerve trifurcation with a 5/0 silk suture and severed distal to the ligation. 0.5cm of the nerve was removed to prevent spontaneous regeneration. Following surgery, anaesthesia was reversed by subcutaneous injection of atipamezole (1mg/kg). The animals were given time to recuperate in a warming chamber before being returned to their home cages. Animals were monitored closely during the recovery period for any signs of discomfort. All efforts were made to minimize pain and discomfort and to reduce the number of animals used. Animals were killed postoperatively at indicated timepoints to collect the ipsilateral and contralateral (control) L4–L5 DRGs.

### Microarray analysis

In three independent experiments control or axotomised L4–L5 mouse DRGs were collected (n = 12–15 in each experiment) and pooled together for RNA extraction. Total RNA was extracted using the miRVANA™ miRNA isolation kit following the manufacturer's instructions (Ambion, UK). Microarray experiments were performed by LC Sciences (Houston, USA; http://www.lcsciences.com). Purified small RNAs were labelled with Cy3 or Cy5 fluorescent dyes and hybridised to dual-channel microarray µParaflo™ microfluidic chips comprising 375 probes that detect unique mature miRNA transcripts listed in the Sanger miRBase Release 9.0 (catalogue number MRA-1002 version number miRMouse_9.0_UB_070209). Fluorescence images were collected using a laser scanner (GenePix 4000B, Molecular Device) and digitized using Array-Pro image analysis software (Media Cybernetics). Data were analyzed by first subtracting the background and then normalizing the signals using a LOWESS filter (Locally-weighted Regression). Following background subtraction and normalisation with Lowess filter, the Cy5/Cy3 ratios were log2-transformed and p-values of the t-test were calculated; differentially detected signals were those with less than 0.05 *p*-values. Array analysis was carried out using TIGR Multi Experiment Viewer (http://www.tm4.org/). Raw data is presented in Table S1. All data is MIAME compliant and has been deposited in GEO under accession number GSE29026 (http://www.ncbi.nlm.nih.gov/geo/).

### qRT-PCR analysis of miRNA

Total RNA from control and axotomised L4–L5 DRGs was purified using the miRVANA™ miRNA isolation kit following the manufacturer's instructions (Ambion, UK). RNA was quantified using a ND-1000 spectrophotometer (NanoDrop Technologies, UK). qRT-PCR analysis of miRNA expression was carried out using Taqman miRNA assays (Ambion, UK) according to manufacturer's instructions and performed on Applied Biosystems 7500 Real-Time PCR System (Applied Biosystems, UK). As an internal control, RNU6B primers were used for RNA template normalization. Experiments were performed in triplicate.

### *In situ* hybridisation

Fresh frozen rat DRGs were sectioned at 10 µm using a cryostat (Cryostat Leica CM3050, UK) and mounted onto Superfrost slides (BDH, UK). Cryosections were fixed in 4% paraformaldehyde and treated with proteinase K (Sigma, UK). The sections were acetylated in acetic anhydride/triethanolamine and then washed in PBS-T. Sections were prehybridised in hybridisation solution for 2hrs at 50°C (50% formamide, 5X saline sodium citrate (SSC), 0.5 mg/ml yeast tRNA, 1X Denhardt's solution). The sections were then hybridised with a DIG-labelled probe complementary to rat miR-21 (0.5 pmol, LNA miRCURY probe; Exiqon UK) overnight at 50°C. Scrambled probes were used as a control. Probe sequences are as follows: miR-21 TCAACATCAGTCTGATAAGCTA; scrambled GTGTAACACGTCTATACGCCCA. Following hybridisation, sections were washed with SSC and incubated at room temperature with blocking solution (60mins, Roche, UK), followed by a second block in 10% normal goat serum (60mins, PBS-T). Sections were incubated with a mouse anti-DIG horseradish peroxidise antibody (1∶500, Abcam, UK) and one of either a rabbit anti-NF200 (1∶1000) or a rabbit anti-CGRP (1∶4000) in a humidified chamber at 4°C overnight. Following PBS-T washes, the sites of NF200 or CGRP antibody labelling were revealed using the secondary antibody Alexa-555 goat anti-rabbit (1∶200, Molecular Probes UK, 120min). Slides were then thoroughly washed in PBS-T (4X 15mins). The *in situ* hybridisation signals were detected using a tyramide amplification system labelled with Alexa-488 according to the manufacturer's instructions (Invitrogen, UK). Slides were washed (PBS-T, 3X 5mins) and mounted in Vectashield mounting medium with DAPI (Vector Labs, UK) before being analysed with a fluorescent microscope (Leica DMRB, UK). For size profiling, 350 miR-21 expressing neurons were measured using ImageJ analysis (http://rsbweb.nih.gov/ij/, NIH).

### Construction and preparation of lentiviral vector overexpressing miR-21 and Spry2

The transcript for miR-21 was PCR purified from mouse genomic DNA and inserted under the control of the CMV promoter in lentiviral vector transfer plasmid (illustrated in Fig. S1). The primers used were 5′ ACTCGAGTCTAGATTGGCATTAAGCCCCAG 3′ and 5′ ACTCGAGGGATCCTCCAAGTCTCACAAGAC 3′. The GFP reporter was tagged at the 3′ end of the miR-21 gene. The cDNA for rat Spry2 was PCR purified from rat spinal cord cDNA using the primers 5′ ATGGAGGCCAGAGCTCAG 3′ and 5′ CTATGTCGGCTTTTCAAAGTTC 3′. The Spry2 cDNA was inserted between the CMV promoter and an IRES-GFP element in the lentiviral vector transfer plasmid (Fig. S1). Lentiviral vectors pseudotyped with the VSVg coat were produced using the triple plasmid transient transfection protocol as previously described [47]. The vector supernatant was concentrated 2000-fold by ultracentrifugation and the resultant vector stocks were titred by FACS analysis of human embryonic kidney HEK293T cells.

### Assessment of neurite outgrowth in DRG neurons

Dissociated adult DRG cultures were prepared as described previously [13]. Neurons were transduced at a multiplicity of infection (MOI) of 10 at days *in vitro* (DIV) 0. At DIV3, neurons were fixed in 4% paraformaldehyde and immunocytochemistry was performed using β-III tubulin (Covance, UK) and GFP (Abcam, UK), followed by detection with Alexa Fluor 488 and Alexa Fluor 546 (Molecular Probes, UK). Neurite outgrowth analyses were carried out by two experimenters independently. Neurites were observed under a fluorescent microscope (Leica DMRB, UK) with representative photomicrographs being captured throughout. To minimize biased quantification, neurite measurements for the first 150–220 transduced neurons encountered when scanning the slide in a systematic manner (regardless of size and number of neurites) were determined using the tracing tool in the plug-in NeuronJ from the ImageJ analysis program (http://rsbweb.nih.gov/ij/, NIH).

### Western blot analysis

Cultured DRG neurons transduced with lentiviral vectors expressing GFP, miR-21 or a control miRNA miR-376b. 3 days after transduction, neurons were lysed with RIPA buffer containing protease inhibitors (Roche, UK). 20 µg protein was separated by gel electrophoresis on 10% gels, transferred to nitrocellulose membranes and detected by immunoblotting using a chemiluminescence system (GE Healthcare, UK). Antibodies that were used for immunoblotting were SPRY2 (Millipore, UK), PTEN (Cell Signalling Technology, UK) and α-tubulin (Sigma, UK). The Western blots were scanned and quantified using National Institutes of Health software ImageJ (http://rsbweb.nih.gov/ij/).

### Luciferase reporter assays

The 3′ UTR regions of mouse and rat Spry2 gene were PCR amplified from mouse and rat genomic DNA respectively using the following primers (lower case represents cloning sites introduced into the PCR product):

Mouse Spry2 UTR: 5′ atacactagtCATTATTAATCAGGAATATTG 3′

5′ atatacgcgtTAATTTCAAACGTTAAT 3′

Rat Spry2 UTR: 5′: 5′ agctactagtCGTTATTAATCAGGAATAC 3′

5′ acgtacgcgtATAAAGGTTGAATTTTAC 3′

Purified PCR products were cloned downstream of the firefly luciferase gene in the pGL3 plasmid and subsequently used in luciferase reporter assays. 40,000 Hela cells were transfected with 100ng of the luciferase reporter, 400ng of plasmid expressing GFP or miRNA and 50ng of pRL-TK control vector encoding Renilla luciferase (Promega, UK) using Fugene (Roche, UK). Firefly and Renilla luciferase activities were measured consecutively in dual-luciferase assays (Promega, UK) 48 hours later.

### Statistical analysis

Data presented as bar graphs are the means ± s.e.m. of at least three independent experiments. Statistical analysis was performed using Student's t-test unless indicated otherwise.

## Supporting Information

Figure S1 **Lentiviral vector overexpressing miR-21.** (a) Schematic diagram of lentiviral vector construct overexpressing miR-21 under the control of the cytomegalovirus (CMV) promoter. (b) HEK293T cells transduced with lentiviral vectors overexpressing GFP only (GFP) or miR-21 at an MOI of 10. Transduced cells were detected by GFP fluorescence. Scale bar represents 100 µm. (c) Quantitative real-time reverse transcription PCR for miR-21 was performed using a Taqman miRNA assay kit (Ambion) to detect overexpression of miR-21 in HEK293T cells. miR-21 expression was normalised to that of the U6B small nuclear RNA gene (RNU6B). Asterisk indicates significant difference in miR-21 transduced cells compared to GFP controls. **p<0.01, Students' t-test, n = 3. (d) Schematic diagram of lentiviral vector construct overexpressing rat SPRY2 under the control of the cytomegalovirus (CMV) promoter. (e) In HEK293T cells that were transduced with the SPRY2-overexpressing lentiviral vectors (MOI of 10), abundant overexpression of SPRY2 was detected by Western blot analysis. Fold changes in SPRY2 expression are normalised to α-tubulin and expressed as a fraction of the levels in untransduced cells, which are assigned a value of 1.00. Fold change in SPRY2 is reflected in bar chart. Asterisk indicates significant difference in SPRY2 neurons compared to controls. *** *p*<0.001, Students' t-test, n = 3.
(TIF)

Table S1 **Microarray analysis of miRNA expression in control and axotomised L4 and L5 DRGs in 3 independent experiments.**
(XLS)
