# Supplementary figures and images for: Axotomy-Induced miR-21 Promotes Axon Growth in Adult Dorsal Root Ganglion Neurons
Source: PLoS One. 2011 Aug 10;6(8):e23423. doi: 10.1371/journal.pone.0023423 (PMC3154476; doi:10.1371/journal.pone.0023423)

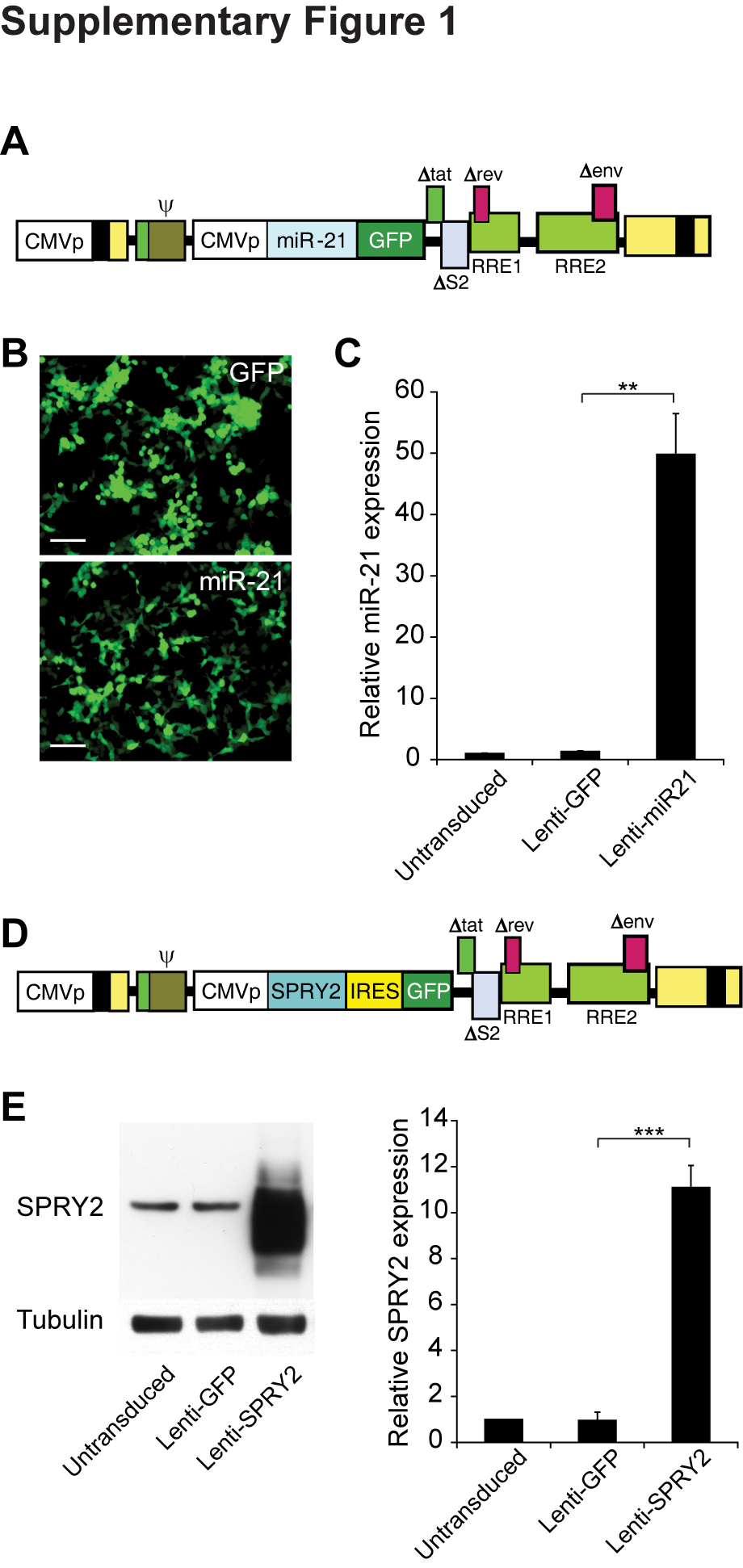

Supplement: Figure S1 — Lentiviral vector overexpressing miR-21. (a) Schematic diagram of lentiviral vector construct overexpressing miR-21 under the control of the cytomegalovirus (CMV) promoter. (b) HEK293T cells transduced with lentiviral vectors overexpressing GFP only (GFP) or miR-21 at an MOI of 10. Transduced cells were detected by GFP fluorescence. Scale bar represents 100 µm. (c) Quantitative real-time reverse transcription PCR for miR-21 was performed using a Taqman miRNA assay kit (Ambion) to detect overexpression of miR-21 in HEK293T cells. miR-21 expression was normalised to that of the U6B small nuclear RNA gene (RNU6B). Asterisk indicates significant difference in miR-21 transduced cells compared to GFP controls. **p<0.01, Students' t-test, n = 3. (d) Schematic diagram of lentiviral vector construct overexpressing rat SPRY2 under the control of the cytomegalovirus (CMV) promoter. (e) In HEK293T cells that were transduced with the SPRY2-overexpressing lentiviral vectors (MOI of 10), abundant overexpression of SPRY2 was detected by Western blot analysis. Fold changes in SPRY2 expression are normalised to α-tubulin and expressed as a fraction of the levels in untransduced cells, which are assigned a value of 1.00. Fold change in SPRY2 is reflected in bar chart. Asterisk indicates significant difference in SPRY2 neurons compared to controls. *** p<0.001, Students' t-test, n = 3. (TIF) [file pone.0023423.s001.tif]
